# Interactions between dorsal and ventral streams for controlling skilled grasp

**DOI:** 10.1016/j.neuropsychologia.2015.07.010

**Published:** 2015-12

**Authors:** Vonne van Polanen, Marco Davare

**Affiliations:** a Motor Control Laboratory, Movement Control and Neuroplasticity Research Group, Biomedical Sciences Group, Department of Kinesiology, KU Leuven, Tervuursevest 101, 3001 Leuven, Belgium; b Sobell Department of Motor Neuroscience and Movement Disorders, UCL Institute of Neurology, University College London, Queen Square, WC1N 3BG London, United Kingdom

**Keywords:** Hand, Grasp, Dorsal stream, Connectivity, Visual input, Haptics

## Abstract

The two visual systems hypothesis suggests processing of visual information into two distinct routes in the brain: a dorsal stream for the control of actions and a ventral stream for the identification of objects. Recently, increasing evidence has shown that the dorsal and ventral streams are not strictly independent, but do interact with each other. In this paper, we argue that the interactions between dorsal and ventral streams are important for controlling complex object-oriented hand movements, especially skilled grasp. Anatomical studies have reported the existence of direct connections between dorsal and ventral stream areas. These physiological interconnections appear to be gradually more active as the precision demands of the grasp become higher. It is hypothesised that the dorsal stream needs to retrieve detailed information about object identity, stored in ventral stream areas, when the object properties require complex fine-tuning of the grasp. In turn, the ventral stream might receive up to date grasp-related information from dorsal stream areas to refine the object internal representation. Future research will provide direct evidence for which specific areas of the two streams interact, the timing of their interactions and in which behavioural context they occur.

## Highlights

• The dorsal and ventral streams are both involved in skilled grasping movements.
• Ventral areas feed dorsal areas with information about object identity.
• Grasps of increased complexity require gradually higher recruitment of ventral areas.
• Dorsal stream inputs could fine tune object representations stored in ventral areas.

## 1 Introduction

When grasping an object, the processing of sensory information is crucial to perform the movement accurately. For instance, the grasp needs to be adapted to the size and location of the object, which can be visually perceived before the start of the motion and implemented into the motor plan. During the movement, visual and haptic feedback can be used to adjust errors. It has been suggested that the information used to control movements is processed differently than information that is used to recognise objects. An influential theory divides the processing of visual information into two streams that follow different routes in the brain after the primary visual cortices. Ungerleider and Mishkin (Mishkin et al., 1983; Ungerleider and Mishkin, 1982) made this division based on anatomical findings in monkeys. From the early visual areas, the ventral stream runs to the inferotemporal cortex, whereas the dorsal stream projects to the posterior parietal cortex. Mishkin et al., (1983) concluded that the ventral stream was important for the identification of objects (‘what’) and the dorsal stream for spatial information (‘where’). Similar dissociations have been found in auditory (Romanski et al., 1999) and haptic (Reed et al., , 2005) perception. Goodale and Milner (Goodale et al., 1994, Goodale and Milner, 1992, Milner and Goodale, 2008) distinguished the two streams not into the kind of information that is processed, but into the function the information is used for. They argued that the ventral stream is involved in the perception of information about objects (vision for perception) and the dorsal stream processes information to guide actions (vision for action). Furthermore, it has been suggested that the dorsal stream can be subdivided into a dorsolateral and dorsomedial circuit (Binkofski and Buxbaum, 2013, Grafton, 2010, Rizzolatti and Matelli, 2003). The dorsolateral circuit includes the anterior intraparietal sulcus (aIPS) and the ventral part of the premotor cortex (PMv). The dorsomedial circuit runs through V6A and the medial intraparietal sulcus to the dorsal premotor cortex (PMd). While dorsomedial regions classically contribute to the planning of reaching movements (Davare et al., 2015, Davare et al., 2012, Vesia and Crawford, 2012), dorsolateral areas integrate grasp-related information (Davare et al., 2007, Davare et al., 2010, Tunik et al., 2005). Recently, these two subcircuits have also been found to interact depending on the degree of online control required by the action (Grol et al., 2007, Verhagen et al., 2013).

Since the introduction of the dual-stream theory, many studies have confirmed and disproved hypotheses of this theory. It is not the scope of this paper to provide an extended review of the literature, as others have provided such overviews (Cloutman, 2013, Grafton, 2010, Milner and Goodale, 2008). Critical views on the theory have been reported as well (Pisella et al., 2006, Schenk and McIntosh, 2010, Smeets and Brenner, 2006). Still, the dual-stream theory remains to have a strong impact on the interpretation of many motor and perceptual findings as it often provides a good fit to the different pattern of results found in motor control or perceptual tasks (e.g. Aglioti et al., 1995). Recently, more attention has been focussed on the possible interactions between the dorsal and ventral streams. This paper proposes that these interactions might be especially important for complex object-oriented hand movements, such as skilled grasp. Skilled grasp can be defined as hand movements requiring independent control of each finger, which has been shown to rely on a highly developed corticospinal tract (Lemon, 2008). The motor command mediating skilled grasp is characterised by fractionated movements and the control of small muscle groups in a highly selective manner. Skilled grasp is driven by sensorimotor processing of object properties, such as the orientation, size, material or shape. In addition, the context in which the object is grasped also plays a role, for instance in the use of tools or when the same object can be used for different applications.

## 2 Anatomical connections between dorsal and ventral streams

Neuroanatomical studies have not only traced the dorsal and ventral pathways (Mishkin et al., 1983; Ungerleider and Mishkin, 1982) but have also established numerous connections between them (see also Cloutman, 2013; Grafton, 2010). In studies where tracer fluids were injected in the brain of monkeys, connections between inferotemporal areas of the ventral stream and parietal areas of the dorsal stream were discovered. For instance, inferotemporal area TE has connections with the intraparietal sulcus and prefrontal areas (Borra et al., 2010). TE was also found to be connected directly to the inferior parietal lobe, or indirectly via the superior temporal sulcus (Zhong and Rockland, 2003). The posterior portion of the inferior temporal cortex TEO has connections with the lateral intraparietal area (Distler et al., 1993). In turn, aIPS has been found to be connected not only to dorsal stream areas, but also to the superior and middle temporal gyrus (Borra et al., 2008).

In human subjects, diffusion tensor imaging reveals white matter tracts between the middle temporal gyrus (MTG) and the supramarginal gyrus in the context of tool use (Ramayya et al., 2010). When applying transcranial magnetic stimulation (TMS) to parietal areas, remote activations have been found in the ipsilateral middle temporal and fusiform gyri (Zanon et al., 2010). Altogether this indicates that both streams are able to communicate with each other in a bidirectional way, although the functional role of the interactions between these separate areas is still unclear.

## 3 Behavioural studies in patients and healthy subjects

Anatomical evidence for connections between the dorsal and ventral streams does not inform us about the behavioural context in which these interactions might be important. One way to address this issue is to investigate what behavioural parameter is impaired when one of the streams is damaged. Indeed, a substantial piece of evidence for the distinct role of the dorsal and ventral streams comes from patient studies. If either the dorsal or ventral stream is damaged, this leads to dissociable behavioural deficits. For example, patients with optic ataxia have lesions in parietal areas, which are part of the dorsal stream. They have deficits in reaching and grasping objects, but are able to visually discriminate different objects. On the other hand, patients with visual form agnosia show lesions to ventral stream areas but have intact dorsal areas. A well-known patient with this type of impairment is patient DF. In contrast to patients with optic ataxia, she is unable to visually identify objects, but if asked to pick them up she performs as well as healthy subjects (Goodale et al., 1994). This pattern of results confirms the hypothesis of the dual-stream theory: the dorsal stream is used to guide actions and the ventral stream to perceive object properties.

Despite this seemingly clear evidence for an independent processing of information, there may be particular action contexts in which retrieving details about object identity is crucial for controlling how the object should be grasped, for example, with tools. The objects that had to be picked up in the study of Goodale et al. (1994), were meaningless shapes. In more demanding tasks such as grasping complex shapes (Goodale et al., 1994), when predictions must be made about tool use (Carey et al., 1996) or comfortable end-position (Dijkerman et al., 2009), errors are seen in DF’s grasping behaviour as well. In addition, lesions in both parietal and occipitotemporal areas are especially associated with impairments in (pantomimed) tool use (Ambron et al., 2015, Hoeren et al., 2014). It is suggested that increased functional interactions between the two streams are needed as the task requires more complex processing of the object conceptual knowledge.

In behavioural studies involving healthy participants, some findings show that the dorsal and ventral streams are not as independent as might have been suggested by the dual-stream theory (Franz et al., 2000, Lee and van Donkelaar, 2002, Pavani et al., 1999, for reviews see Schenk and McIntosh (2010), Smeets and Brenner, (2006) and Smeets et al. (2002). For example, illusions thought only to influence the ventral stream (e.g. Aglioti et al., 1995) have been found to affect the lifting, but not the grasping of objects (Brenner and Smeets, 1996). Furthermore, a visually noticeable weight change after the start of the movement can be incorporated into the lifting movement of an object, despite the short time frame (Brouwer et al., 2006). The authors argued that this online adjustment, thought to rely on the dorsal stream, seems to be influenced by visual cues about weight information that are processed by the ventral stream.

Although behavioural studies provide valuable insights into the functioning of the brain, it cannot be inferred which specific areas of each stream interact. The question might not be *if* the two streams interact, but rather *how*, *when,* and in which behavioural context. As mentioned above, the various anatomical sources for connections between areas of the dorsal and ventral streams do not reveal how these areas communicate in a specific task. Patient studies have the limitation that lesions are often incomplete or spread over multiple areas. Therefore, patients show a variety of impairments (Pisella et al., 2006), where it is not always clear if these deficits arise from damages in lower or higher order visual areas (Serino et al., 2014). In addition, redundancy and adaptation of specific areas might lead to false conclusions. Non-invasive procedures like transcranial magnetic stimulation (TMS), electroencephalograms (EEG) and functional magnetic resonance imaging (fMRI) allow us to study the activation of specific brain areas in healthy subjects. For instance, during grasping movements, an extensive network of cortical areas is recruited (Binkofski et al., 1999, Ehrsson et al., 2000, Gallivan et al., 2011, for reviews see also Davare et al., 2011 and Grafton, 2010). There is, however, little research that looked at the involvement of both dorsal and ventral stream areas in grasping. In the following section, we will discuss fMRI and TMS studies investigating skilled grasping behaviour in healthy participants.

## 4 Interactions between dorsal and ventral stream subserve skilled grasp

The dorsal stream is involved in the control of action with online visual feedback. When the object must be grasped from memory, as happens when a delay is introduced between the presentation of the object and the actual grasp, online visual control is not possible. As such, delayed grasping is thought to be controlled by the ventral stream (Milner and Goodale, 2008). Indeed, optic ataxia patients improve in the pointing to targets and grasping if a delay is introduced (Milner et al., 2001, Milner et al., 1999), whereas for visual agnosia patient DF performance decreases (Goodale et al., 1994).

In contrast to this idea, other studies have shown that *both* the dorsal and ventral streams are involved in delayed grasping. Cohen et al. (2009) applied TMS to the lateral occipital cortex (LO), a brain area in the ventral stream, and the aIPS, a dorsal stream area. They found that TMS to LO impaired delayed grasping, but TMS to aIPS impaired both immediate and delayed grasping. In an fMRI study, Singhal et al. (2013) found that the lateral occipital complex (LOC) and the early visual cortex (EVC) were reactivated with delayed grasping. Dorsal stream areas remained active during the delay, as shown by sustained activation of aIPS, PMd and the supplementary motor area (SMA). These studies indicate that in delayed or memory driven tasks the ventral and dorsal areas interact to control grasping. The information that is needed to perform an accurate grasping movement when no visual input is present anymore (i.e. after the delay) seems to be acquired from ventral stream areas.

Moreover, in grasping experiments without a delay, circumstances arise where the interactions between dorsal and ventral streams are more important. In a study of Verhagen et al. (2008), participants were asked to grasp an object that was positioned in different orientations with monocular or binocular vision. When the object is more slanted, the depth information from pictorial cues becomes more important in monocular viewing conditions. They found that in this condition the ventral stream area LOtv was more coupled with dorsal stream areas aIPS and PMv. The authors concluded from this that when prehension relies on pictorial information, the ventral stream supports the dorsal stream in the organisation of the movement.

An important limitation of the above studies is that the object was not visible during grasping (Verhagen et al., 2008) or even during the planning of the grasping movement (Cohen et al., 2009, Singhal et al., 2013) therefore preventing online visual control. Moreover, there are indications that in studies using remembered targets it is not the delay or a shift from dorsal to ventral stream control, but the amount of visual feedback that causes differences between grasping after a delay and with full vision (Franz et al., 2009). Since the dual-stream theory states that the dorsal stream controls movements through online visual control, it remains unclear if the same results would be found under full vision. Some experiments with sustained visual information have been performed with pantomime grasping and tool use. In pantomime grasping, increased connections between aIPS and the posterior inferotemporal gyrus (pITG) were found when participants had to change their grip selection for an object (Makuuchi et al., 2012). The authors used a dynamic causal model analysis for the pantomimed grasping of different objects with a precision or power grip. However, it must be noted that pantomimed grasping recruits different brain areas than actual grasping (Króliczak et al., 2007).

In even more complex tasks than simple grasping, like tool use, a broad network of brain areas is active. Brandi et al. (2014) showed that when objects were used instead of just transported, dorsomedial areas are recruited, but also ventral LOC. Specifically for tool use compared to the use of neutral bars, dorsolateral areas are more active as well as the MTG in the ventral stream. This suggests that tool use, which requires complex movements and conceptual knowledge of the objects, critically relies on interactions between ventral and dorsal areas.

It is noteworthy that besides interconnections between different areas of both streams, there might also be single areas that are involved in object identification as well as motor control processes. For example, motion processing area V5/MT which is active in the perception of motion (Maunsell and Van Essen, 1983) was also found to be involved in catching a moving object compared to grasping a stationary object (Schenk et al., 2005).

A recent study examined the representation of object weight in dorsal stream areas controlling object lifting and ventral stream areas processing object properties (Gallivan et al., 2014). The authors focussed on the primary motor cortex, the somatosensory cortex and PMd, as well as ventral areas in LOC: LO and the posterior fusiform sulcus. Interestingly, LOC represented object weight, even though no visual differences were visible between the objects. When there were different texture cues, texture-sensitive areas in the occipitotemporal complex (OTC) represented object weight as well as texture. These results indicate that properties that are not visual and related to object-oriented actions are represented in the ventral stream. The ventral stream could thus be involved in storing learned associations between visual object properties and mechanical properties, for example weight, that can be used to guide behaviour.

In sum, there are situations in which the dorsal and ventral streams seem to be jointly involved in grasping. The ventral stream seems to be gradually more recruited as information about the object from pictorial cues or memory is needed to control the grasping movement, or if conceptual knowledge about more complex objects that are used every day or tools needs to be retrieved for allowing the most appropriate grasp.

## 5 Towards defining a possible hierarchical organisation in ventral-dorsal stream interactions

Still much remains unknown about the precise interactions between the dorsal and ventral streams underlying grasping (see also Cloutman (2013) for several options). First, how these areas are hierarchically linked remains an open question. Besides anatomical connections, little is known about which areas do functionally interact and which areas control how much interaction is needed between the two streams. Regarding the input of the ventral to the dorsal stream, the more lateral part of the dorsal stream might play a specific role, as the dorsolateral stream is suggested to be more involved in using rather than just grasping objects (Binkofski and Buxbaum, 2013) and considering its anatomically more adjacent position to the ventral stream, it is plausible that information from the ventral stream is fed to the dorsolateral stream. Could the reliance on object identity information in more complex object-oriented hand actions be processed by the dorsolateral stream without inputs from the ventral stream? This seems unlikely considering the finding of the involvement of LOC, which is part of the ventral stream. A candidate structure in the dorsal stream that might combine information from the ventral stream in sensorimotor control tasks is aIPS. This area has connections with many other areas in both streams (Borra et al., 2008) and can incorporate spatial and pictorial information into the motor plan (Verhagen et al., 2012). The involvement of aIPS in cross-modal (visual and haptic) processing of object features (Grefkes et al., 2002) might also be relevant for this integration, considering the importance of haptic feedback for the calibration of grasping movements (Bingham et al., 2007, Schenk, 2012). Moreover, LOC plays a role in object recognition for both haptically and visually perceived stimuli (Amedi et al., 2001, Costantini et al., 2011). The multimodal nature of these areas might be a benefit for complex grasping tasks. In other words, increased interactions between, for instance, LOC and aIPS might be important to integrate information from multiple senses into the grasp plan.

How information arising from the two streams is finally implemented into a grasping plan is another open question. Top-down control and recurrent processes might arrange the information that is gathered from earlier visual areas. The dorsal stream projects to premotor areas PMv and PMd, which as mentioned earlier, play a significant role in grasping (Davare et al., 2006, Singhal et al., 2013, Verhagen et al., 2008) and can bias the motor command. For instance, in an experiment where monkeys had to reach for targets, neuronal recordings indicated that PMd was activated before the parietal reach region (PRR) when monkeys were free to choose the target compared to a predefined search order, suggesting that PMd influenced PRR when a decision for selecting a movement had to be made (Pesaran et al., 2008). Likewise, prefrontal areas might play an important part in structuring the incoming information from the two streams. In the affordance competition hypothesis, information is processed recurrently in parietofrontal loops until enough evidence has been gathered for selecting a specific action amongst competing alternatives (Cisek, 2007). For example, it has been shown that the dorsolateral prefrontal cortex (DLPFC) is involved in action selection, especially when individuals are free to choose their movement (Duque et al., 2010, Hadland et al., 2001, Hasan et al., 2013) Here we argue for a similar mechanism in selecting the appropriate grasp amongst multiple competing grasps driven by the combination of an object representation and its contextual use located in ventral stream areas. The role of DLPFC in skilled grasp is not yet understood but it is likely DLPFC channels the interactions between the ventral and dorsal stream based on how context should be incorporated into the selection of a specific grasp.

This hierarchical organisation of ventral-dorsal stream interactions can also control *when* these connections come into play. Information transfer from the ventral stream into the motor command that is implemented by dorsal stream areas might occur in a gradual way. Fast movements might be controlled automatically (e.g. automatic pilot, Pisella et al., 2000), whereas for movements in which (non-spatial) object properties must be integrated in the movement or movement correction input from the ventral stream is required, but this may take time. A study in which participants had to make pointing movements to different targets has found that the improved behaviour when pointing with a delay was gradual with optic ataxia patients (Himmelbach and Karnath, 2005). This suggests a continuous transfer from dorsal to ventral stream control. Milner and Goodale (2008) mention that there might be a distinction between the planning and programming of an action, as the planning might involve receiving information about object properties from the ventral stream, whereas the dorsal stream conducts the programming. However, ventral stream activations have been found during the execution phase of a grasping movement as well (Brandi et al., 2014, Singhal et al., 2013). Unfortunately, subtle timing differences are difficult to distinguish in fMRI experiments. Methods with a higher temporal resolution could provide more insight into the relative timing of activation between ventral and dorsal areas.

Similar to a possible gradual transfer of information from the ventral stream to the dorsal stream as a function of time, there might also be a continuous increase of interactions between the streams with increasing task complexity. Ranging from simple grasping tasks to complex tool use, where conceptual knowledge about object functions and thus identity is more important, an increase of ventral stream contribution might be expected. Depending on the task context, information from the ventral stream might be weighted differently in a way comparable to maximum-likelihood estimation (Ernst and Banks, 2002). In this model, sensory information from multiple sources is incorporated into the estimation of an object property based on its reliability, with higher weights given to more reliable sensory sources. In a similar way, the impact on grasp control can be higher when specific object identity information is more relevant and reliable for the task. This weighting might present itself especially in illusionary contexts, where information conflicts occur. In addition, time and accuracy constraints could shape the ventral stream input, because these computations are likely to increase processing time in the typically fast dorsal stream visuomotor loops. Furthermore, experience with the task might alter the contribution weighting. The gradual interaction between ventral and dorsal stream might be altered if a movement is well learned and becomes automatic. Milner and Goodale (2008) argued that unpractised movements required influence of the ventral stream that transferred to dorsal stream control during learning. However, when contextual information is still important, ventral stream influence will probably not be completely diminished. For instance, a highly practiced movement like picking up a cup of tea still requires knowledge about whether the cup is made out of plastic or porcelain to lift it with the correct force. Ventral-dorsal interactions might be more optimized in these perfected and familiar actions, possibly involving memorized motion schemas that are learned to be associated with specific contextual information. Therefore, experience might also decrease time costs associated with slow ventral stream processing by shaping connectivity strength as more permanent pathways are formed.

A largely unaddressed point is the transfer of information from the dorsal stream to the ventral stream. The study of Gallivan et al. (2014) suggests that information gathered from a grasp (weight) can be stored in the ventral stream, possibly to make associations between mechanical and material object properties. In this way, action related information can be used to build up a richer internal object representation. These bidirectional interactions between the ventral and dorsal streams might be crucial for learning to manipulate new objects.

Finally, a more difficult question is *what* kind of information is transferred. It might seem inefficient to process each object property twice: once in the ventral and once in the dorsal stream (Franz et al., 2009). Similarly, different effects of illusions on motor and perceptual tasks might be explained by different attributes that are used to perform the task (Smeets et al., 2002). It is difficult to infer what information is used or even necessary to perform certain tasks, but investigating the interactions in specific task contexts might lead to a better understanding of the information that is communicated between the different areas in the two streams.

## 6 Conclusions

We suggest that the dorsal and ventral streams alone are not sufficient to control skilled grasp, but that interactions between the streams are necessary. In simple action contexts, grasp control can be driven only by the dorsal stream, but as the complexity of object-related properties increases and information about object identity is necessary, the involvement of ventral stream areas becomes more prominent for ensuring accurate movement performance. Still, much about the nature, strength and timing of interactions between both streams remains unknown and further research is needed to address these issues.

## Acknowledgements

VVP is funded by a Fonds Wetenschappelijk Onderzoek grant to MD (FWO Odysseus, Belgium). MD is funded by a BBSRC (BBSRC: BB/J014184/1) David Phillips fellowship (UK) and a Fonds Wetenschappelijk Onderzoek grant (FWO Odysseus, Belgium) (FWO: G/0C51/13N).
